# Effective surgical outcomes of the pararectus approach for psoas abscess: a case series

**DOI:** 10.1093/jscr/rjaf114

**Published:** 2025-03-07

**Authors:** Yi-Jou Chen, Yi-Hsun Yu

**Affiliations:** Department of Orthopedic Surgery, Musculoskeletal Research Center, Chang Gung Memorial Hospital, Linkou Branch, Chang Gung University, Taoyuan, Taiwan; Department of Orthopedic Surgery, Musculoskeletal Research Center, Chang Gung Memorial Hospital, Linkou Branch, Chang Gung University, Taoyuan, Taiwan

**Keywords:** soft tissue infection, surgical approach, debridement

## Abstract

A psoas abscess is a rare but potentially life-threatening condition that requires timely diagnosis and management. Surgical intervention is essential in patients with percutaneous drainage failure, multiloculated abscesses, or implant-related pathologies. This case series highlights the use of the pararectus approach, originally developed for acetabular fractures, to manage complex retroperitoneal infections. Two cases are presented: a metastatic breast cancer patient with a left psoas abscess extending to the hip joint and a patient with a history of pelvic and spinal instrumentation presenting with bilateral iliopsoas abscesses. Both patients underwent successful surgical debridement using the pararectus approach, demonstrating its efficacy in accessing and draining retroperitoneal abscesses, with minimal complications. This approach provides an effective visualization of critical structures, enabling comprehensive treatment while minimizing tissue damage. These findings underscore the utility of the pararectus approach for managing challenging psoas abscesses, ensuring rapid drainage and bacterial culture acquisition for targeted antimicrobial therapy.

## Introduction

A psoas abscess is a rare but potentially life-threatening condition characterized by pus collection within the iliopsoas compartment. It may result from a primary hematogenous infection or be secondary to adjacent infections, including postsurgical complications [1, 2]. Its nonspecific presentation, including fever, back pain, and limp, often delays the diagnosis and increases morbidity and mortality. Management typically involves antimicrobial therapy and drainage, with surgical debridement being necessary in cases of percutaneous failure, multiloculated abscesses, or orthopedic implant-related pathology [2, 3].

The pararectus approach, originally developed for acetabular fractures, offers minimally invasive access to the retroperitoneal space and iliopsoas muscle [4]. Extensive experience using this approach for pelvic ring and acetabular fractures has shown that it provides straightforward access to the iliopsoas compartment [4, 5]. Here, we present two cases of psoas abscesses that were successfully treated with surgical debridement via the pararectus approach, highlighting its effectiveness in managing complex retroperitoneal infections.

## Case series

### Case 1

**Figure 1:**
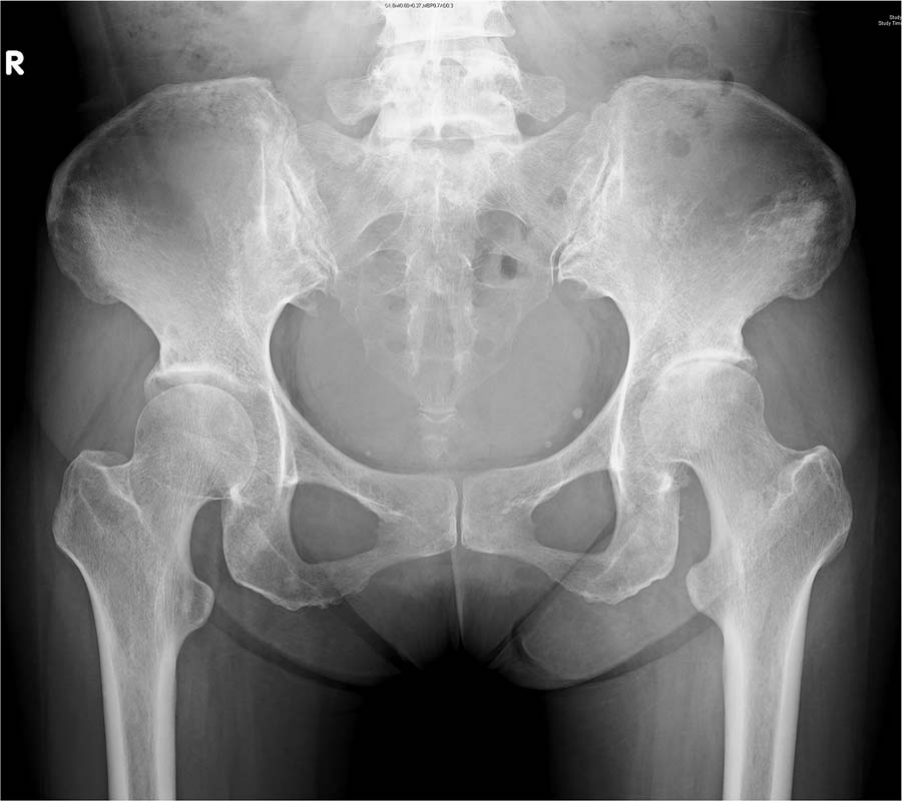
Pelvic radiograph of a 44-year-old female showing symmetric left hip joint space narrowing, indicative of hip septic arthritis.

**Figure 2:**
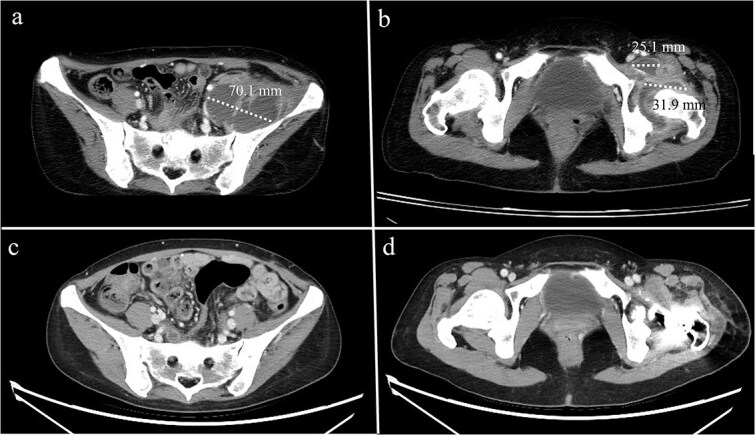
Computed tomography of a 44-year-old female demonstrating (a) a psoas abscess and (b) hip septic arthritis. Postdebridement surgery and resection arthroplasty, the abscess completely resolved (c, d).

A 44-year-old female, with a medical history of invasive ductal carcinoma of the breast with multiple sites of metastasis (brain, pulmonary, liver, and bone), presented to the outpatient clinic with progressive intractable pain over the left inguinal area in the preceding month. Radiography revealed symmetrical joint space narrowing of the left hip joint (Fig. 1). Computed tomography (CT)revealed a well-lobulated cystic lesion within the left psoas muscle, extending caudally into the hip joint (Fig. 2a and b). The serum C-reactive protein (CRP) level was 55.04 mg/dl at admission. Due to the inaccessibility of CT-guided percutaneous abscess drainage, she underwent a scheduled surgical debridement of the psoas abscess. Resection arthroplasty with polymethylmethacrylate hip spacer implantation was planned simultaneously.

**Figure 3:**
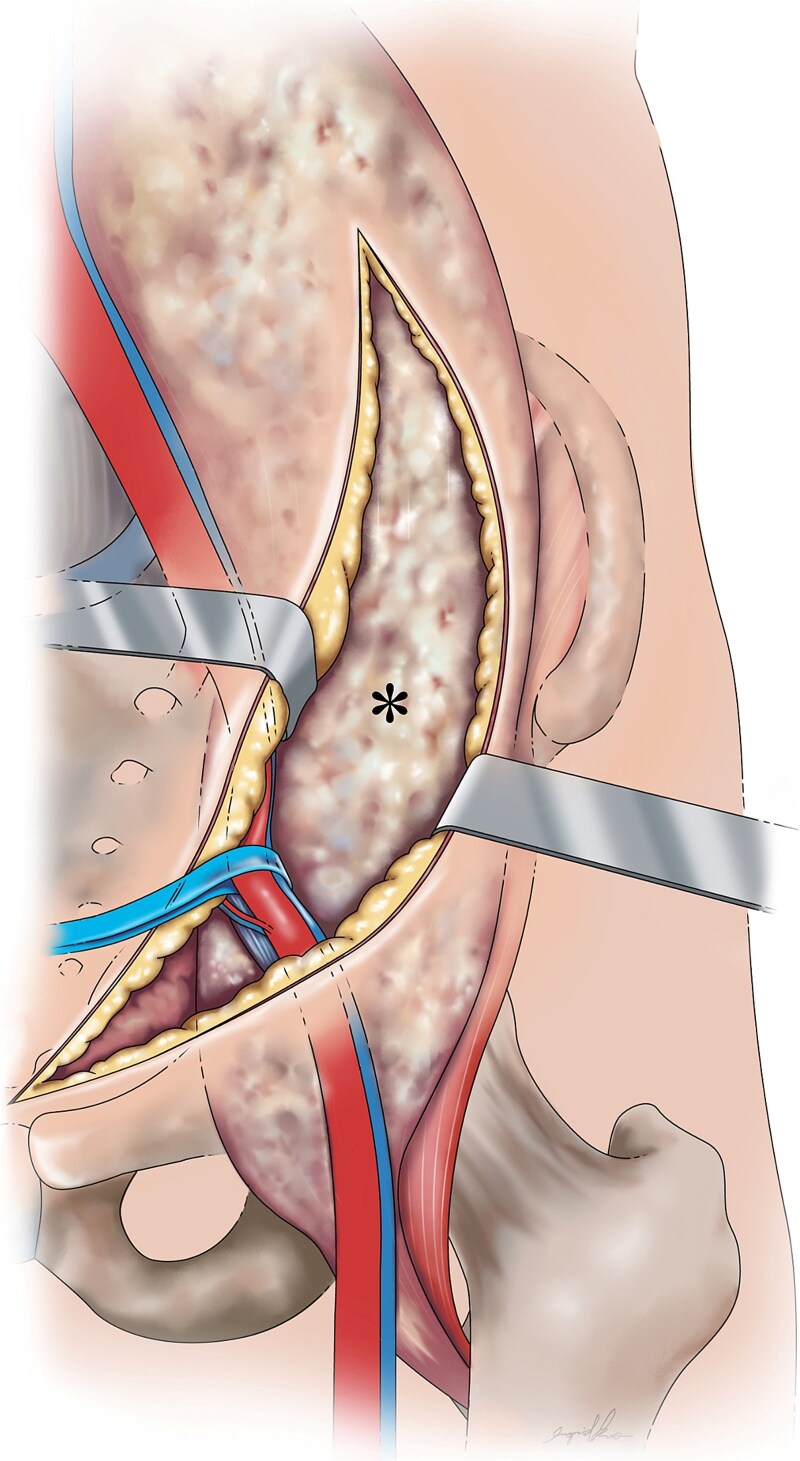
Graphical depiction of a psoas abscess (*) following dissection from the abdominal wall, fascia layers (Camper’s and Scarpa’s fascia), external oblique aponeurosis, and transversalis fascia with medial retraction of the external iliac vessels.

The surgery consisted of two stages: the pararectus approach for the psoas abscess and the modified Hardinge approach for the hip joint. During the pararectus approach, the involved iliacus psoas was detected by blunt dissection after surgical dissection, deepening into the external abdominal aponeurosis (Fig. 3). Approximately 400 ml of purulent fluid was drained. After confirming that there was no retained lobulated abscess within the retroperitoneum, the surgical wound was closed in layers, and two Jackson-Pratt drains were inserted. Following this procedure, the hip lesion was treated as scheduled.

**Figure 4:**
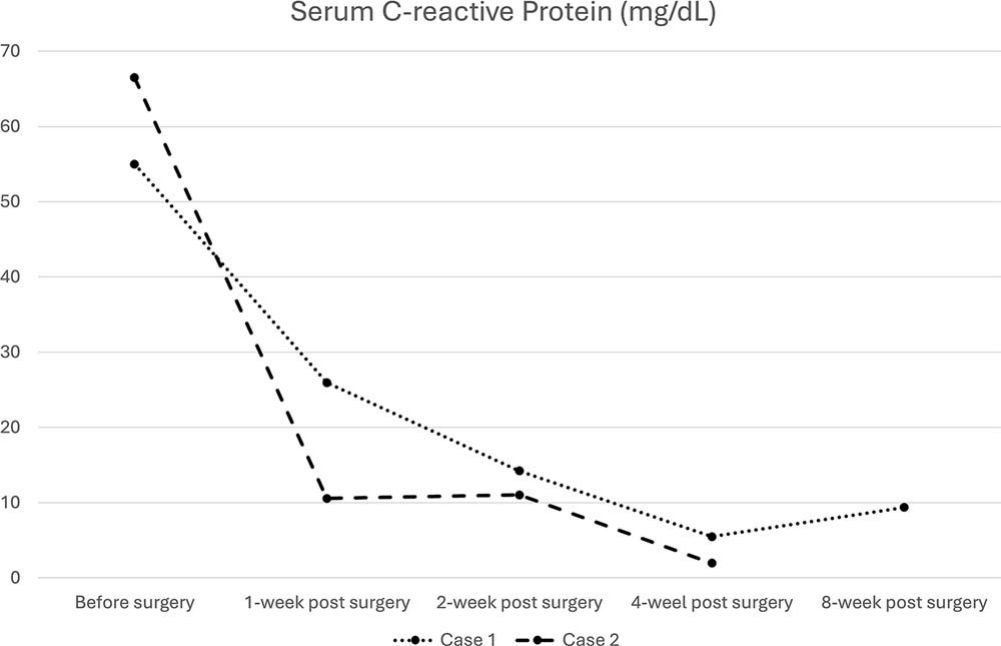
Serial serum C-reactive protein measurements for both cases.

A bacterial culture of the pus yielded *Staphylococcus aureus*. After debridement surgery and parental use of sensitive antimicrobial agents, the CRP levels decreased over time (Fig. 4). A repeat CT scan revealed a completely diminished abscess within the iliac fossa (Fig. 3c and d). The patient underwent total hip arthroplasty three months after the first surgery. However, she died 2 months later because of multiple organ failure caused by cancer metastasis.

### Case 2

**Figure 5:**
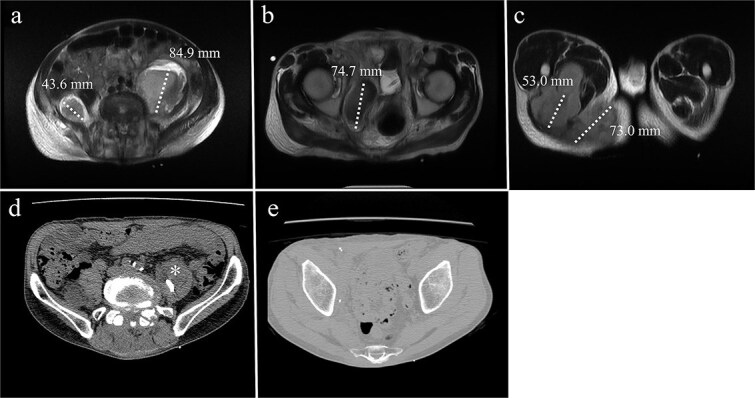
MRI of a 68-year-old male with (a) bilateral psoas abscesses (right: 43.6 mm, left: 84.9 mm). The right abscess extended caudally to (b) the quadrilateral plate of the acetabulum and (c) the medial thigh and ischial tuberosity region. (d) Partial resolution of the right abscess with persistence of the left abscess (*). (e) Complete resolution of the right caudal extension after debridement.

A 68-year-old male with a history of pelvic ring injury (fixation with two iliac screws 10 years prior) and degenerative lumbar spondylosis (spinal instrumentation 1 year prior) presented to the emergency department with fever and buttock pain. Magnetic resonance imaging revealed bilateral lobulated abscesses in the iliopsoas muscle (Fig. 5a–c). However, the abscess in the right psoas muscle extended caudally to the ischial tubercle and adductor muscles of the right thigh. Due to persistent fever with intractable pain in the right lower abdomen and right thigh, surgical debridement of the right psoas abscess was planned. The left psoas muscle was drained using a percutaneous CT-guided procedure because it was a solitary lesion.

**Figure 6:**
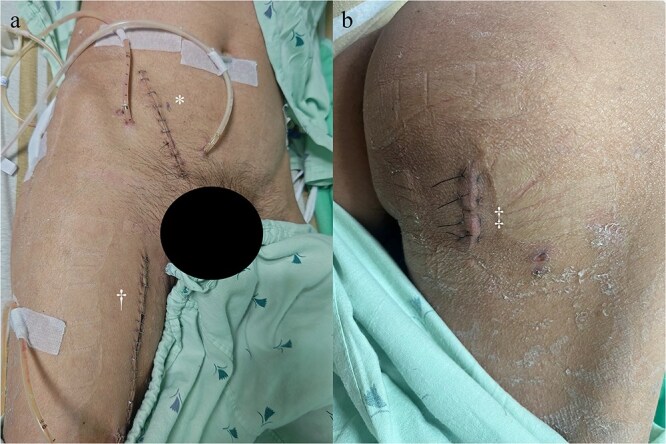
Surgical incisions: (a) pararectus incision (*) and medial thigh incision (†). (b) Skin incision (‡) for ischial tuberosity abscess drainage.

The surgical procedure using the pararectus approach was similar to that used in Case 1. Surgical debridement was extended to the medial thigh and posterior buttocks (Fig. 6). Approximately 300 ml of pus was drained during surgical debridement. The bacterial culture yielded *Streptococcus pyogenes*. Serum CRP levels decreased progressively after surgery (Fig. 3). A repeat CT scan revealed a small residual abscess over the right iliac fossa with complete resolution of the infection of the left thigh and posterior buttocks (Fig. 5c and d). However, the resolution process was slow in the left psoas abscess treated with percutaneous drainage.

## Discussion

A psoas abscess is a challenging condition that requires timely intervention. Although parental antibiotics and percutaneous drainage are often effective, surgical debridement is essential in complex cases [6, 7]. For patients with rapidly progressing life-threatening sepsis and undetermined bacterial species, surgical intervention provides benefits in the rapid evacuation of the abscess and selection of sensitive antimicrobial agents based on the results of bacterial culture.

The goal of surgery is to effectively drain the abscess and remove the infected tissue while minimizing damage to the surrounding structures. Surgical debridement using an endoscopic or laparoscopic approach provides the advantage of a minimally invasive intervention to reduce postoperative pain [3, 7]. However, retroperitoneal tissue adhesion due to the infection process may be dangerous owing to limited endoscopic vision. Furthermore, debridement may be insufficient for lobulated abscesses. Open surgical drainage may involve an anterior or posterior approach, depending on the location of the abscess, and is often performed with combined spinal instrumentations [8–10]. Unfortunately, these approaches may not be able to detect intrapelvic infectious lesions, resulting in inadequate treatment.

The iliac fossa approach [11], also referred to as the lateral window of the ilioinguinal approach, has traditionally been used for open debridement in such cases. This approach is primarily favored for its safety, as it avoids critical vascular and neural structures. However, the operative field provided by this approach is limited to the area beneath the iliacus muscle, whereas the psoas muscle, which is located more medially, is less accessible. Consequently, this often results in an increased likelihood of missing the target lesion or incomplete debridement.

Contrastingly, the pararectus approach facilitates comprehensive debridement through a minimally invasive surgical incision, offering effective access to the iliopsoas muscle and enabling prompt abscess drainage. This approach ensures thorough treatment while minimizing complications. Typically, a surgical incision measuring 6–8 cm is sufficient to identify critical anatomical structures, including the inferior epigastric vessels, spermatic cord or round ligament, and external iliac vessels, and to explore the infected iliopsoas muscle. Furthermore, the surgical field can be readily extended proximal to the iliosacral joint or distal to the retropubic space, depending on the extent of the abscess.

In conclusion, when a psoas abscess is indicated for surgical intervention, the pararectus approach offers effective and limited surgical intervention with rapid draining of the retroperitoneal abscess and obtaining bacterial pathology.
